# Development and clinical validation of an ERA-CRISPR/Cas12a assay for the rapid detection of 14 high-risk HPV types

**DOI:** 10.1128/spectrum.03036-25

**Published:** 2025-12-26

**Authors:** Zhijie Wang, Ting Hu, Wanxin Liu, Hu Zhou, Xinyi Lv, Hui Li, Xuemeng Li, Xiaoyuan Huang, Liang He

**Affiliations:** 1 Department of Obstetrics and Gynecology, Tongji Hospital, Tongji Medical College, Huazhong University of Science and Technology 12443, Wuhan, Hubei, China; 2 National Clinical Research Center for Obstetrics and Gynecology, Cancer Biology Research Center (Key Laboratory of the Ministry of Education), Tongji Hospital, Tongji Medical College, Huazhong University of Science and Technology 12443, Wuhan, Hubei, China; 3 Wuhan Kandwise Biotechnology, Inc. Wuhan, Hubei, China; Children's National Hospital, George Washington University, Washington, DC, USA

**Keywords:** cervical cancer, human papillomavirus/ HPV, ERA, HR-HPV detection, CRISPR/Cas12a

## Abstract

Persistent infection with high-risk human papillomavirus (HR-HPV) is the leading cause of cervical cancer, highlighting the critical need for early detection to improve prevention. Although real-time quantitative polymerase chain reaction (RT-qPCR) remains the gold standard for HR-HPV detection, its dependence on sophisticated equipment, complex procedures, and trained personnel limits accessibility. Here, we developed a simplified assay for 14 HR-HPV types by integrating direct lysis, enzyme-mediated isothermal rapid amplification (ERA), and CRISPR-Cas12a-mediated cleavage into a streamlined workflow that requires only a basic isothermal heating device. The optimized system achieved a sensitivity of 50 copies per reaction with no cross-reactivity, while a refined lysis buffer containing 20% Chelex-100 minimized inhibition from vaginal swab samples, thereby enhancing detection performance. Validation with 152 clinical samples demonstrated 97.62% sensitivity and 100% specificity, confirming the reliability of the method. This user-friendly and cost-effective assay requires minimal equipment, enabling rapid and field-deployable HR-HPV detection, and offers a practical alternative to conventional laboratory-based approaches, particularly in resource-limited settings.

**IMPORTANCE:**

High-risk human papillomavirus (HR-HPV) is the principal etiological agent of cervical cancer, and early detection remains central to effective disease prevention. Current PCR-based assays, however, rely on specialized laboratories and trained personnel, limiting their deployment in many settings. Here, we report a streamlined CRISPR-Cas12a assay that integrates direct sample lysis, ERA, and CRISPR-based detection into a single workflow operable with only a simple heating device to determine the presence of 14 HR-HPV types. The assay achieves high analytical sensitivity, strong specificity, and robust clinical performance while maintaining low cost and ease of use. This platform enables rapid HR-HPV detection and scalable screening, particularly in resource-constrained environments, with the potential to facilitate earlier intervention and reduce cervical cancer incidence.

## INTRODUCTION

In 2022, cervical cancer was the fourth most common cancer among women globally, with approximately 660,000 new cases and 350,000 deaths (1). The incidence and mortality rates for cervical cancer vary significantly between countries. In developing countries, the incidence is 19.3 per 100,000 women, and mortality is 12.4 per 100,000, while in developed countries, these rates are 12.1 per 100,000 and 4.8 per 100,000, respectively (1). The World Health Organization’s global Cervical Cancer Elimination Initiative aims to reduce incidence rates to below 4 per 100,000 women-years by the end of this century, ultimately eliminating cervical cancer as a public health problem (2). We are now in a critical phase of accelerating the elimination of cervical cancer, and improving screening coverage with high-quality methods is one of the key strategies to achieve this goal (3).

Cervical cancer has a well-established etiology, with the majority of cases caused by human papillomavirus (HPV) infection (4). HPV is a common sexually transmitted infection that affects the skin, genital area, and throat. It is a small DNA virus with an approximately 8 kb circular double-stranded genome, consisting of the early gene region (E1–E7), the long control region (LCR), and the late gene region (L1 and L2) (5–8). Due to its high conservation across various HPV types, the L1 region is frequently targeted for HPV detection in both clinical and epidemiological studies (9, 10). HPV is classified into low-risk (LR) and high-risk (HR) types based on their potential to cause anogenital cancer (11–14). HR-HPV genotypes, such as HPV 16 and 18, are strongly linked to cervical cancer, and persistent infection can lead to abnormal cell changes that may progress to cancer (15). In contrast, LR-HPV genotypes, such as HPV 6 and 11, mainly cause benign conditions like genital warts and rarely contribute to cancer development (15–18). The WHO’s 2021 cervical cancer screening guideline includes 14 HR-HPV genotypes: HPV 16, 18, 31, 33, 35, 39, 45, 51, 52, 56, 58, 59, 66, and 68 (19). This guideline stresses the importance of efficient HPV screening to detect and manage HR-HPV infections early, which can reduce cervical cancer incidence and mortality. Thus, developing an effective detection method that covers all 14 HR-HPV types is critical for early management of HR-HPV infections, helping to reduce cervical cancer rates. Despite significant progress in HPV screening, several challenges remain. Screening coverage is uneven, especially in low-income countries where limited medical resources contribute to higher cervical cancer rates. Current nucleic acid-based detection methods, such as RT-qPCR, offer high sensitivity and accuracy but require specialized personnel, complex sample preparation, and expensive equipment (20), limiting their accessibility in resource-limited settings. Therefore, there is an urgent need for cost-effective, user-friendly HPV detection tools that do not require complex instrumentation.

In recent years, CRISPR/Cas12 and Cas13, with their trans-cleavage activity, have enabled the development of CRISPR-based molecular diagnostic platforms, such as SHERLOCK (21), HOLMES (3), and DETECTR (22). When target DNA or RNA is present in the sample, CRISPR RNA (crRNA) directs Cas12 and Cas13 to bind selectively to the target DNA or RNA sequence, respectively. This interaction activates the trans-cleavage activity of Cas12 or Cas13, leading to the degradation of single-stranded DNA (ssDNA) or single-stranded RNA (ssRNA) probes, which amplifies the signal for highly sensitive detection (3, 21–24). Combining CRISPR-based detection with isothermal amplification increases sensitivity, reduces assay time, and eliminates the need for thermal cycling, making it suitable for resource-limited environments (25). Notably, lateral flow-based CRISPR detection is cost-effective and does not require fluorescence visualization (26, 27). Despite these advancements, challenges remain in developing CRISPR-based HPV detection systems. Multiplexed isothermal amplification and CRISPR detection may compromise sensitivity; vaginal swab samples contain inhibitory components that can affect direct amplification; and fluorescence-based detection still requires expensive equipment. Therefore, optimizing a simple, cost-effective, and highly sensitive assay that covers all 14 HR-HPV genotypes is essential.

In this study, we developed a rapid, user-friendly, and cost-effective HR-HPV detection system by integrating CRISPR/Cas12a with a direct lysis protocol and enzyme-mediated isothermal rapid amplification (ERA). System optimization enabled single-tube detection of 14 HR-HPV genotypes with a detection limit of 50 copies per reaction, no cross-reactivity, and results delivered within 30 min. Validation with 152 clinical samples demonstrated a clinical sensitivity of 97.6% and specificity of 100%. These results indicate that the CRISPR/Cas12a-based platform is a reliable tool for HR-HPV diagnosis. Its simplicity, rapidity, affordability, and high accuracy make it a promising alternative for expanding HPV screening, particularly in low-resource settings, thereby facilitating early detection and contributing to cervical cancer prevention.

## MATERIALS AND METHODS

### Material

LbCas12a was obtained from Guangzhou Magigen Biotech Co., Ltd. (Guangzhou, China). ERA amplification reagents and extraction-free lysis buffer were purchased from Suzhou GenDx Biotechnology Co., Ltd. (Jiangsu, China). Lateral flow assays (LFA) strips were purchased from Wuhan Shiwei Biotech Co., Ltd. (Hubei, China). HPV genotyping detection kit was acquired from Jiangsu Shuoshi Biotechnology Co., Ltd. (Jiangsu, China). Full-length HPV reference sequence plasmids were synthesized by Nanjing GenScript Biotech Corporation (Jiangsu, China). All primers, crRNAs, and single-stranded DNA reporters (ssDNA reporters) were synthesized by Sangon Biotech Co., Ltd. (Shanghai, China). Chelex 100 was purchased from Sigma-Aldrich, St Louis, MO, USA.

### Methods

#### Design and screening of ERA primers and crRNA

Full-length reference genomes of HR HPV types were retrieved from the Papillomavirus Episteme (PaVE) database (28), which is accessible at https://pave.niaid.nih.gov/. Based on these sequences, plasmids containing the complete reference genomes were synthesized by Nanjing GenScript Biotech Co., Ltd. To target highly conserved regions within the L1 gene of 14 HR-HPV types, three sets of ERA amplification primers were designed and synthesized. Primer efficiency was assessed using the Qsep100 capillary electrophoresis system, with selection criteria based on the prominence of the main peak and alignment of the amplicon size with theoretical values. The optimal primer set was chosen for target gene amplification (Table S1).

After selecting primers, target sites within the amplified sequence that met the TTTN protospacer adjacent motif (PAM) requirement were identified. Based on these sites, three crRNAs were designed for each HR-HPV type (Table S2). HR-HPV plasmids served as detection templates to determine the optimal crRNA. Further validation was conducted using LR-HPV plasmids and common sexually transmitted pathogens, including *Neisseria gonorrhoeae* (NG), *Mycoplasma genitalium* (MG), *Chlamydia trachomatis* (CT), and *Ureaplasma urealyticum* (UU), to evaluate potential cross-reactivity.

#### ERA reaction assay

According to the manufacturer’s guidelines for ERA reactions, each lyophilized ERA reaction pellet contains a complete ERA reaction system. The rehydration mixture was prepared as follows: Forward primer (2.5 μL, 10 μM), Reverse primer (2.5 μL, 10 μM), DNA template (20 μL, with concentration adjusted as needed), ddH₂O (23 μL), Magnesium acetate (2 μL, 280 mM). The mixture was vortexed thoroughly, briefly centrifuged, and then incubated at 40°C for 15 min. Following amplification, the ERA products were either analyzed using different detection methods or stored at −20°C for subsequent Cas12a-mediated cleavage assays.

#### CRISPR/Cas12a cleavage detection

The CRISPR/Cas12a cleavage assay was conducted according to the manufacturer’s instructions. Briefly, the reaction mixture (50 μL total volume) contained 1 μL of LbCas12a (2 μM), 1 μL of crRNA (2 μM), 5 μL of ssDNA reporter (2 μM), 10 μL of 5× cleavage buffer, and 20 μL of sample, with nuclease-free water added to adjust the final volume. The ssDNA reporter was labeled with FAM at the 5′ end and BHQ at the 3′ end for fluorescence detection, or with FAM at the 5′ end and biotin at the 3′ end for lateral flow detection. For fluorescence readout, reactions were incubated at 37 °C and monitored on a QuantStudio 3 Real-Time PCR System (Applied Biosystems, USA) using the FAM channel, with data collected at 1-min intervals. For lateral flow readout, reactions were incubated at 37°C for 15 min, after which the test strip was directly inserted into the reaction tube for visualization.

#### Determination of the limit of detection

The LOD for each HR-HPV genotype in the multiplex detection system was determined using serial dilutions of plasmid standards containing 0, 10, 50, and 100 copies per reaction. For each concentration, plasmid samples representing all 14 HR-HPV genotypes were first amplified using the ERA method. The resulting amplicons were then subjected to CRISPR/Cas12a fluorescence assays. A reaction was defined as positive when the fluorescence signal intensity exceeded twofold that of the negative control. Each concentration was tested in three independent experimental replicates. A sample was considered positive only when all three replicates produced positive signals; otherwise, it was classified as negative.

#### Clinical sample detection

A total of 152 clinical cervical swab samples were collected from Tongji Hospital, affiliated with Tongji Medical College, Huazhong University of Science and Technology, with approval from the hospital’s ethics committee (TJ-IRB202505050). Among these, 126 samples tested positive for 14 HR-HPV subtypes, while 26 samples were 14 HR-HPV-negative, as determined using the HPV genotyping kit from Jiangsu Bioperfectus Technologies Co., Ltd. (Jiangsu, China). All clinical samples were further analyzed using a CRISPR/Cas12a-based detection method.

## RESULTS

### Design and screening of ERA primers and crRNA

The primary objective of this study was to develop a multiplex amplification system for detecting 14 HR-HPV subtypes. To achieve this, three sets of highly conserved primers targeting the L1 region were designed for screening based on the high conservation of the L1 region in HPV (9, 10). The primer sets used for amplification were prepared by equimolar mixing of primers targeting 14 HR HPV types, with a final concentration of 10 μM. The amplification efficiency of the primer sets was evaluated using the Qsep100 capillary electrophoresis system, with assessment criteria including the prominence of the main peak, minimal nonspecific amplification, and concordance of amplicon size with theoretical expectations. Among the tested sets, primer set #1 generated a distinct amplification band with minimal nonspecific products, and the amplicon size was consistent with the predicted length (Fig. S1). Accordingly, primer set #1 was selected for subsequent experiments (Table S1). Based on its amplicons, crRNAs incorporating a PAM sequence (TTTV) were designed, with three crRNAs designed for each target.

**Fig 1:**
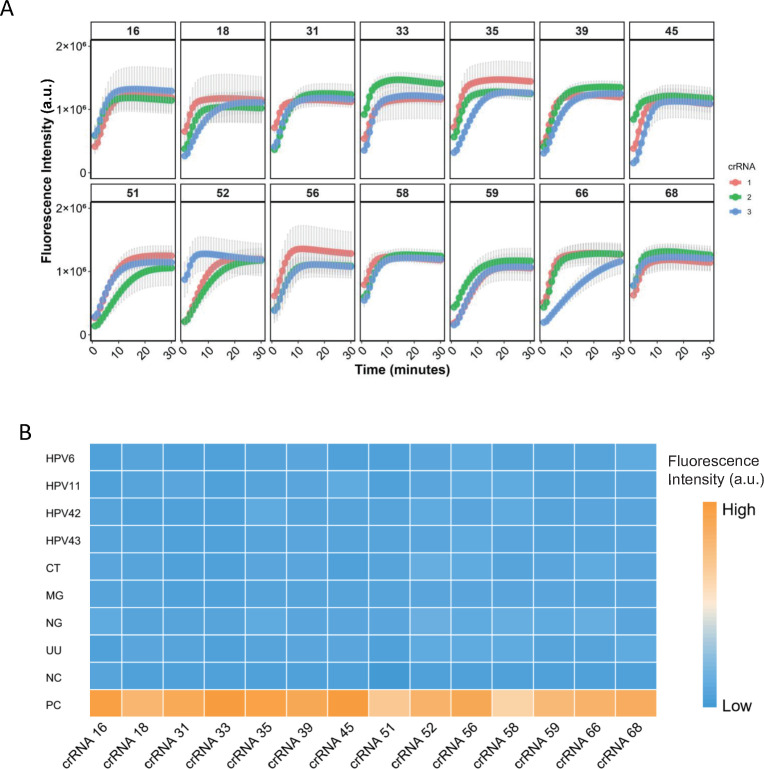
Design and screening of crRNAs. (**A**) Screening of crRNAs targeting 14 HR-HPV types, with three crRNAs designed for each HPV. Plasmids were used individually as templates for Cas12a-based detection. Data are presented as mean ± SE from three independent experiments. (**B**) Specificity assessment using plasmids of common low-risk HPV (LR-HPV) types (HPV6, HPV11, HPV42, HPV43) and DNA from clinically positive samples of common sexually transmitted pathogens (NG, MG, CT, and UU). No cross-reactivity was observed, confirming the high specificity of the detection system. Data are presented as mean from three independent experiments.

To assess the performance of the designed crRNAs, each of the 14 HR-HPV plasmids was used as a template for PCR amplification with primer set #1. The resulting amplicons were subsequently subjected to Cas12a-mediated detection. crRNAs were evaluated based on early fluorescence onset and high signal intensity. As shown in Fig. 1A, the most effective crRNAs were identified as HPV16-crRNA3, HPV18-crRNA1, HPV31-crRNA2, HPV33-crRNA2, HPV35-crRNA1, HPV39-crRNA2, HPV45-crRNA2, HPV51-crRNA1, HPV52-crRNA3, HPV56-crRNA1, HPV58-crRNA2, HPV59-crRNA2, HPV66-crRNA1, and HPV68-crRNA2. These crRNAs exhibited robust target recognition, providing a solid foundation for subsequent assay development.

Further specificity assessments were conducted to evaluate potential cross-reactivity. First, common low-risk HPV (LR-HPV) types outside the detection panel, including HPV6, HPV11, HPV42, and HPV43, were tested. Second, potential interference from common sexually transmitted pathogens—*Neisseria gonorrhoeae* (NG), *Mycoplasma genitalium* (MG), *Chlamydia trachomatis* (CT), and *Ureaplasma urealyticum* (UU)—was examined. For LR-HPV, plasmids containing full-length sequences were used as templates, whereas DNA extracted from clinically positive samples was employed for NG, MG, CT, and UU. Both ERA amplification and CRISPR-Cas12a cleavage assays were performed. As shown in Fig. 1B, positive fluorescence signals were observed exclusively with HR-HPV plasmid templates, while LR-HPV and the tested pathogens produced negligible signals, confirming the absence of cross-reactivity in the detection system.

### Optimization of primer mix ratio and concentration

**Fig 2:**
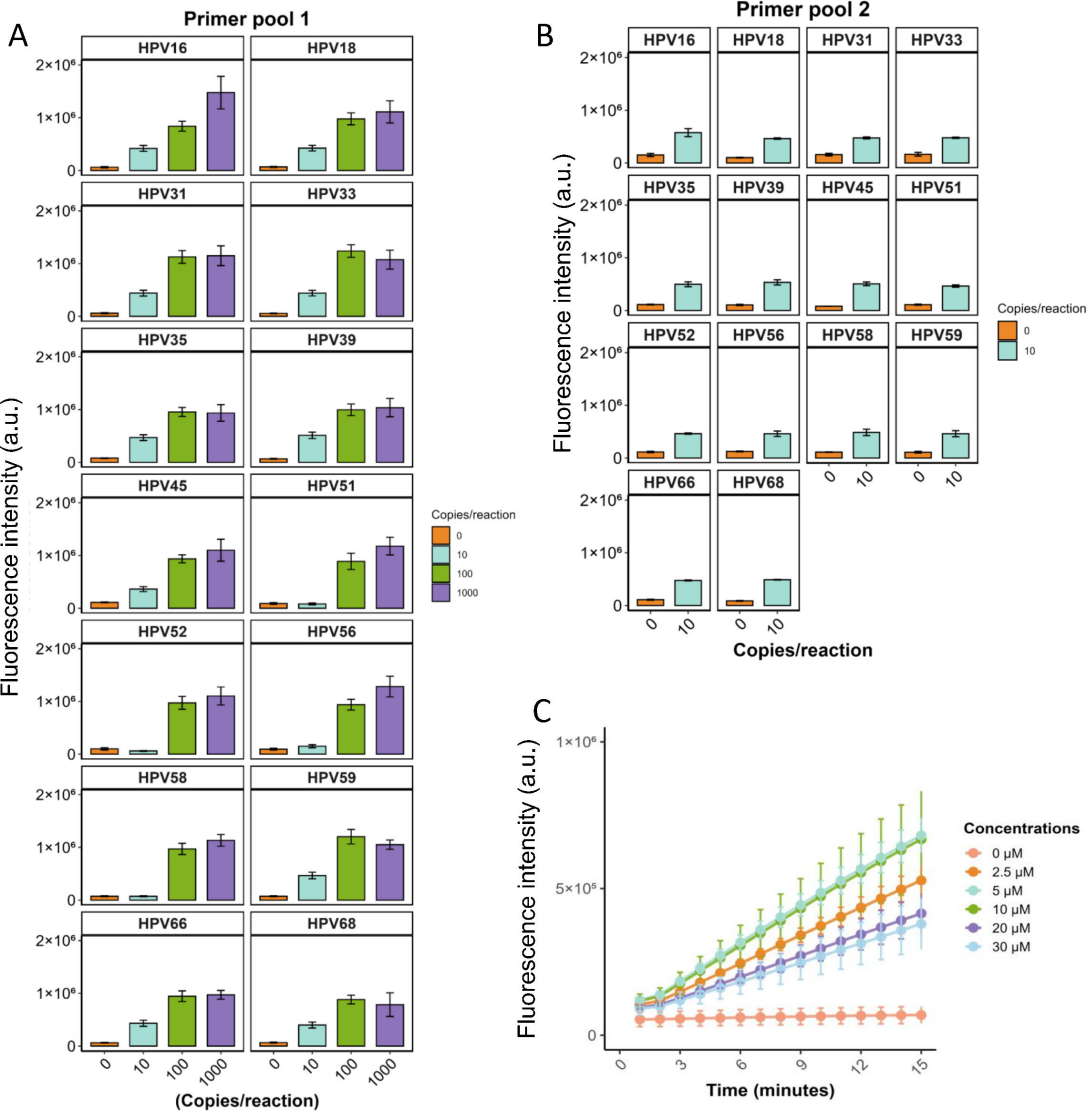
Optimization of primer mix ratio and concentration. (**A**) Amplification of 14 HR-HPV types at 100, 10, 5, and 0 copies per reaction using an equimolar Primer Pool 1, followed by CRISPR-based detection with the corresponding single crRNA. Data are presented as mean ± SE from three independent experiments. (**B**) Amplification at 10 and 0 copies per reaction using Primer Pool 2, in which primers for HPV51, HPV52, HPV56, HPV58, and HPV66 were doubled relative to Pool 1, showing improved fluorescence signals for low-copy templates. Data are presented as mean ± SE from three independent experiments. (**C**) Testing different concentrations of Primer Pool 2 (0–30 μM) using HPV16 (10 copies per reaction) to optimize amplification efficiency and CRISPR-based fluorescence detection. Data are presented as mean ± SE from three independent experiments.

To optimize the primer pool for 14 HR-HPV detections, we conducted a series of evaluations. First, we prepared Primer Pool 1 by combining 14 pairs of HR-HPV primers in equal proportions, resulting in a final total concentration of 10 μM. This primer pool was used for ERA amplification of HR-HPV templates at varying concentrations (0, 10, 100, and 1,000 copies per reaction). The amplification products were then subjected to CRISPR-based signal detection using the optimal single crRNA selected for each HPV type. As Fig. 2A shows, when the template input was 10 copies per reaction, the detection signals for HPV51, HPV52, HPV56, HPV58, and HPV66 were comparable to those of the NC group, whereas the fluorescence signals for the other 10 HR-HPV types were significantly higher than the NC group. To address this, we doubled the proportions of HPV51, HPV52, HPV56, HPV58, and HPV66 primers within primer pool 1, thereby enhancing their relative abundance and yielding primer pool 2. Subsequent testing using Primer pool 2 for amplification of all 14 HR-HPV templates (10 copies per reaction) demonstrated positive detection results across all HR-HPV types (Fig. 2B). Thus, Primer Pool 2 was selected for further detection of the 14 HR-HPV subtypes.

Previous studies have shown that total primer concentration can significantly impact the efficiency of isothermal amplification (29). Following the establishment of the optimal primer ratio, we optimized the total primer concentration by testing a range of 0–30 μM. Using HPV16 (50 copies per reaction) as the template, we observed that amplification efficiency was compromised at both low and high concentrations, whereas the CRISPR assay produced maximal fluorescence at 5 and 10 μM (Fig. 2C). On this basis, we selected 5 μM as the optimal total primer concentration.

### Optimization of crRNA mixing ratio and concentration

**Fig 3:**
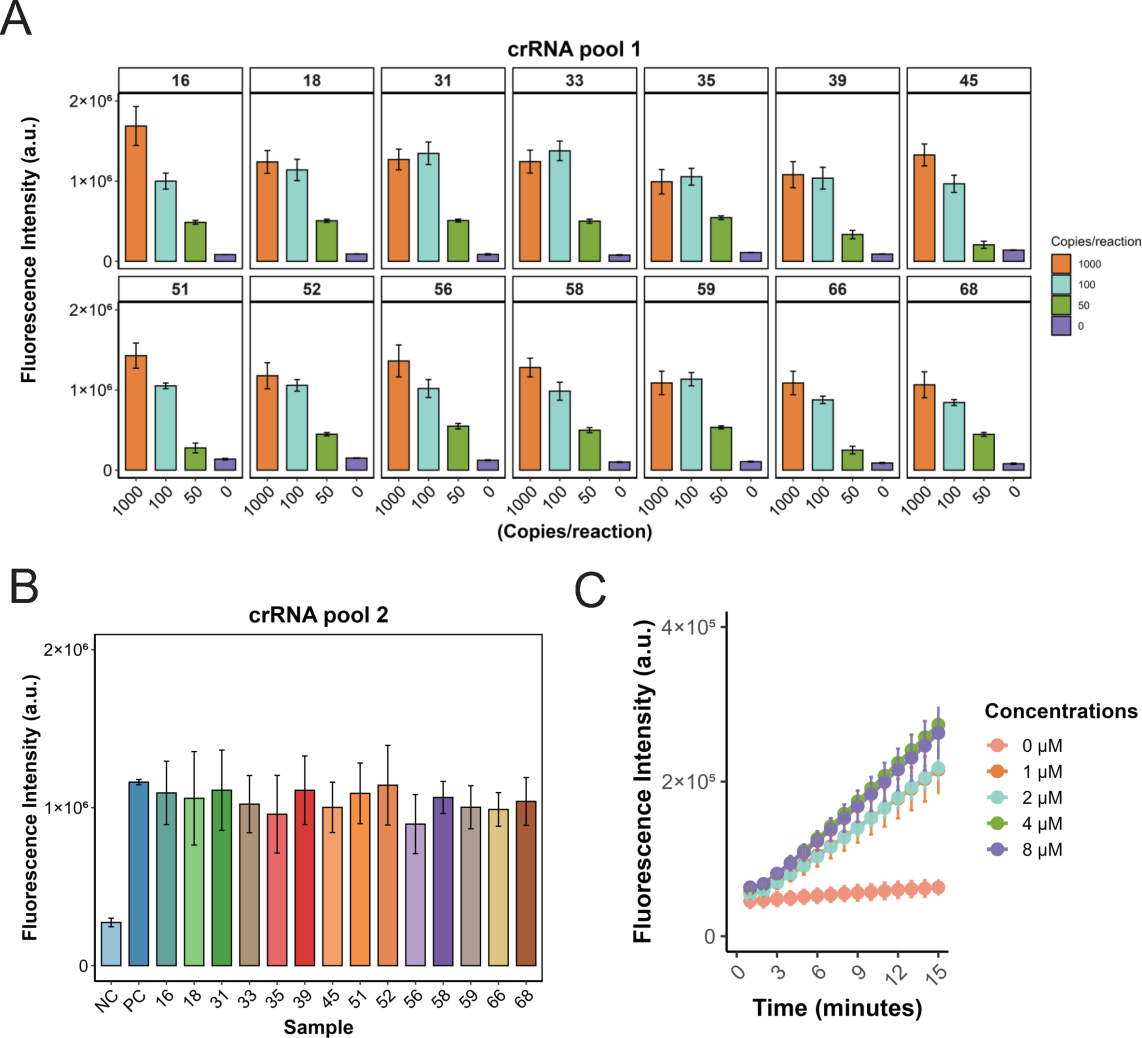
Optimization of crRNA mixing ratio and concentration. (**A**) Amplification of 14 HR-HPV types at 0, 50, 100, and 1,000 copies per reaction using Primer Pool 2, followed by CRISPR-based detection with crRNA Pool 1 to measure fluorescence signals. Data are presented as mean ± SE from three independent experiments. (**B**) Amplification of 14 HR-HPV types at 50 copies per reaction, followed by fluorescence detection using crRNA Pool 2, in which the relative abundance of HPV39, HPV45, HPV51, and HPV66 crRNAs was doubled, enhancing the detection of low-copy templates. Data are presented as mean ± SE from three independent experiments. (**C**) CRISPR-mediated fluorescence detection using HPV16 (50 copies per reaction) amplification products with crRNA Pool 2 at varying concentrations (0, 1, 2, 4, and 8 μM) to optimize crRNA performance. Data are presented as mean ± SE from three independent experiments.

During the previous primer optimization, a single crRNA was used for detection. For simultaneous detection of 14 HR-HPV subtypes, a mixture of different crRNAs is required. We, therefore, optimized the crRNA composition. Using Primer Pool 1 for amplification, detection was evaluated at template inputs of 1,000, 100, 50, and 0 copies per reaction. With crRNA Pool 1, an equimolar mixture of all 14 crRNAs, all HR-HPV subtypes were detectable at 1,000 and 100 copies. However, at 50 copies, four subtypes (HPV39, HPV45, HPV51, and HPV66) exhibited relatively weak fluorescence signals (Fig. 3A). To improve sensitivity at low template input, the proportions of these four crRNAs were doubled, generating an optimized formulation (crRNA Pool 2). This adjustment enabled robust detection of all 14 HR-HPV subtypes at 50 copies per reaction (Fig. 3B).

We also evaluated the effect of total crRNA concentration (0–8 μM) on assay performance. Increasing the concentration above 4 μM did not further enhance the signal (Fig. 3C). Therefore, 4 μM was selected as the optimal crRNA pool concentration.

### Optimizing reaction conditions for the ERA-CRISPR system

**Fig 4:**
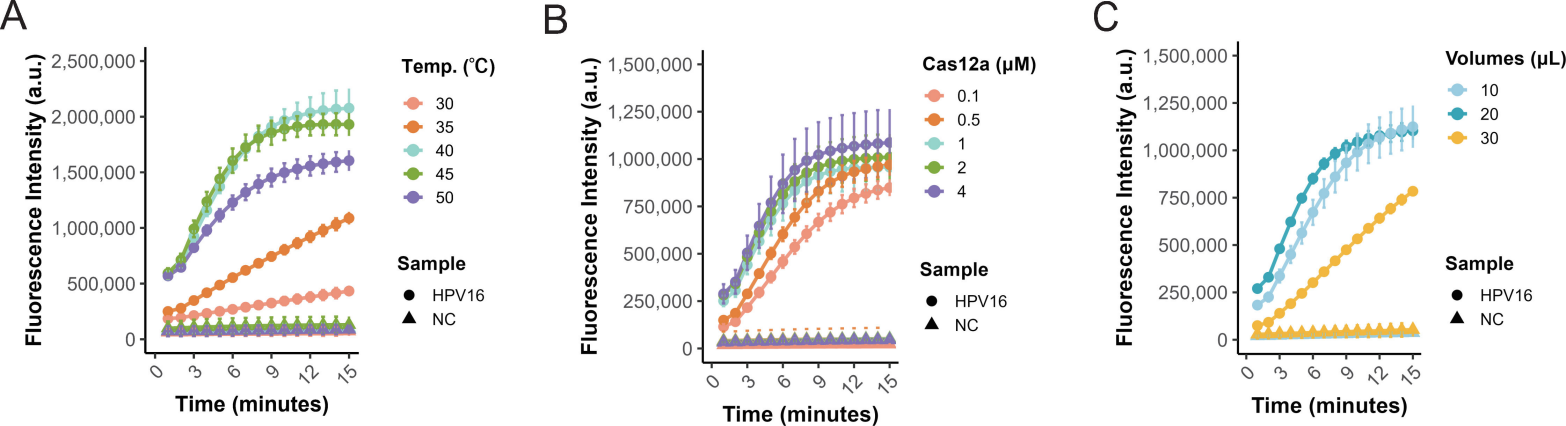
Optimization of ERA-CRISPR/Cas12a reaction conditions. (**A**) Amplification and CRISPR/Cas12a fluorescence detection were assessed at different temperatures (35°C, 40°C, 45°C, 50°C, and 55°C) using HPV16 as the template. Data are presented as mean ± SE from three independent experiments. (**B**) Cas12a enzyme at varying concentrations (0.1, 0.5, 1, 2, and 4 μM) was tested to determine the optimal concentration with HPV16 (50 copies/reaction). Data are presented as mean ± SE from three independent experiments. (**C**) Different volumes of ERA amplification products (10, 20, and 30 μL) were added to a 50 μL CRISPR cleavage reaction to evaluate their impact on cleavage efficiency. Data are presented as mean ± SE from three independent experiments.

The reaction temperature of the ERA-CRISPR system was optimized using HPV16 plasmid (50 copies per reaction) as the template. To enhance practical utility and avoid the need for variable-temperature equipment, both ERA amplification and Cas12a detection were conducted at a uniform temperature. Across a gradient of 30–50°C, efficient detection was observed, with fluorescence peaking at 40°C (Fig. 4A). Thus, 40°C was selected as the optimal reaction temperature.

Cas12a enzyme concentration was subsequently optimized over the range 0.1–4 μM (0.1, 0.5, 1, 2, and 4 μM). Detection performance increased significantly at concentrations ≤ 1 μM, whereas higher concentrations did not further enhance fluorescence signals (Fig. 4B). Accordingly, 1 μM was chosen as the optimal enzyme concentration.

Finally, we investigated the effect of ERA amplification product volume on the Cas12a reaction. ERA products of 10, 20, or 30 μL were individually added to a 50 μL CRISPR reaction. Fluorescence intensity was comparable at 10 and 20 μL, with slightly higher signal at 20 μL, whereas 30 μL led to signal suppression (Fig. 4C), indicating that excessive ERA product can inhibit Cas12a activity. Accordingly, an input volume of 20 μL was selected for downstream reactions.

### Preliminary determination of the limit of detection

**Fig 5:**
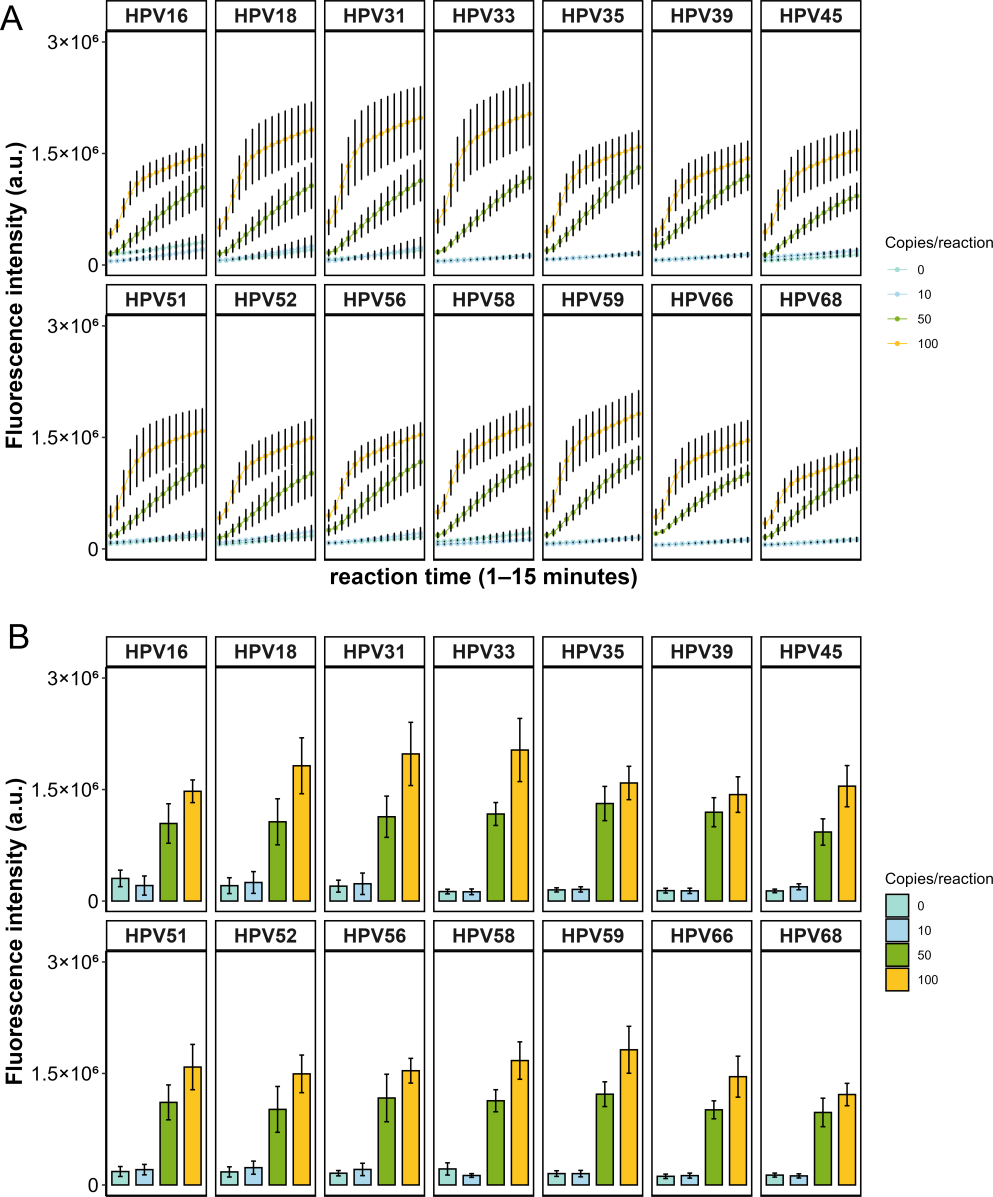
Preliminary determination of the limit of detection. (**A**) Raw fluorescence data collected over 1–15 min for each HR-HPV type at different template concentrations (0, 10, 50, and 100 copies per reaction) using the optimized ERA-CRISPR system. (**B**) Bar plot showing fluorescence intensity at 15 min for each HPV type and concentration. Data are presented as mean ± SE from three independent experiments.

Based on the optimization process, we preliminarily determined the parameters for the HPV multiplex detection system and assessed its LOD. For each HR-HPV type, template DNA was tested at concentrations of 0, 10, 50, and 100 copies per reaction using ERA amplification with Primer Pool 2 (5 μM) at 40°C, followed by CRISPR-based cleavage with Cas12a (1 μM) and crRNA Pool 2 (4 μM) to measure fluorescence signals. Reactions containing 50 or 100 copies per reaction produced positive signals, whereas reactions with 0 or 10 copies per reaction were negative (Fig. 5A and B). We further evaluated the reproducibility of the ERA-CRISPR assay using LOD samples (50 copies per reaction) for all 14 HR-HPV types. Three operators (A, B, and C) each performed three independent batches. For every HPV type, positive samples yielded consistent positive results across operators and replicates, while the negative control remained negative (Fig. S3). The uniform detection pattern across all conditions demonstrates high inter-operator and inter-run reproducibility of the assay. Together, these results indicate that the system’s LOD is 50 copies per reaction (Fig. 5).

### Optimization of extraction-free lysis system

**Fig 6:**
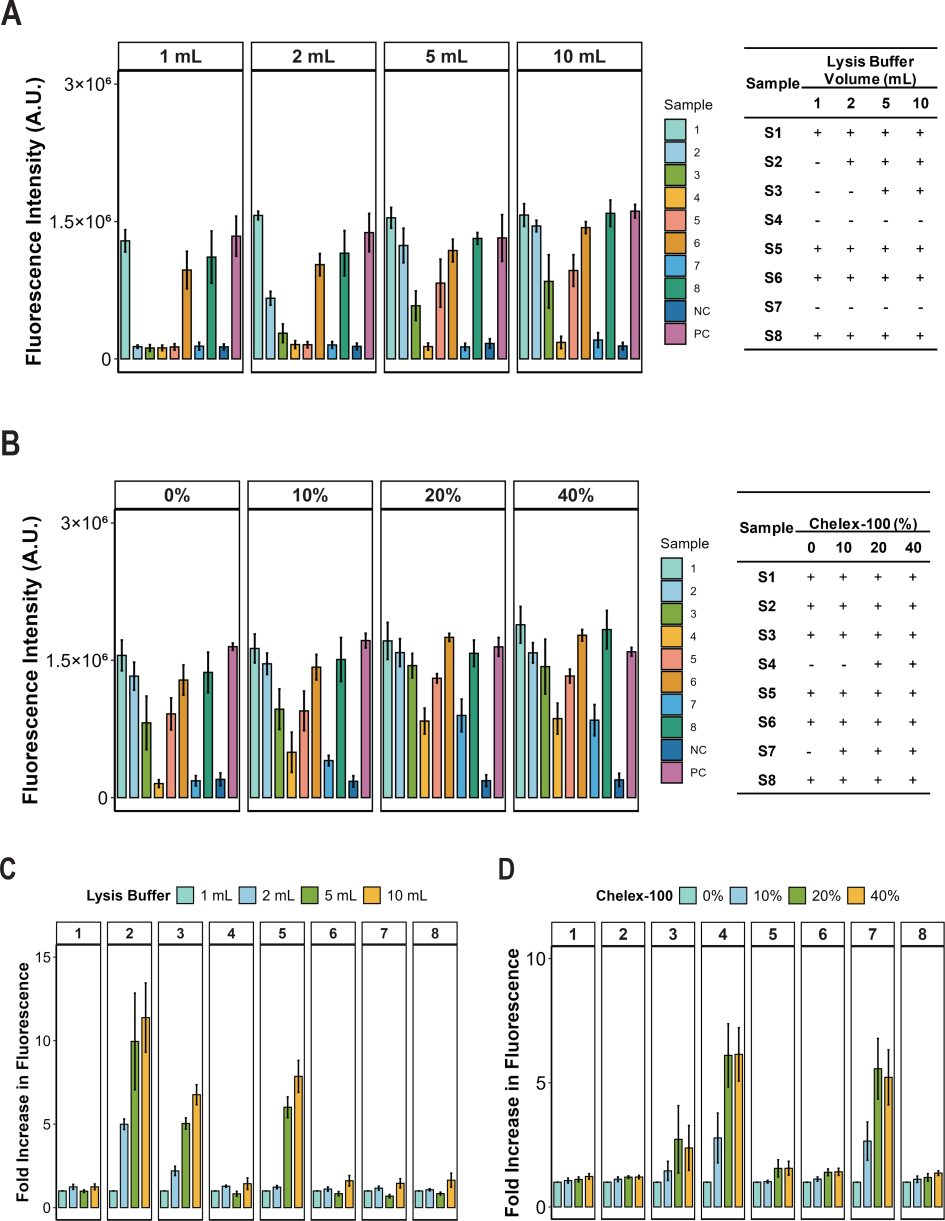
Optimization of extraction-free lysis system. (**A**) Different lysis buffer volumes (1, 2, 5, and 10 mL) were tested with eight clinical samples to determine the optimal volume. Data are presented as mean ± SE from three independent experiments. (**B**) A 5 mL lysis buffer containing Chelex-100 at varying concentrations (0%, 10%, 20%, and 40%) was applied for CRISPR/Cas12a fluorescence detection of eight clinical samples, respectively. (**C**) Fluorescence fold change for eight samples using different volumes of lysis buffer (1, 2, 5, and 10 mL, respectively). (**D**) Fluorescence fold change for eight samples at 10%, 20%, and 40% Chelex-100 concentrations, respectively. Data are presented as mean ± SE from three independent experiments.

Through the above optimization process, we established our HR-HPV detection system using plasmid samples as detection templates, achieving a preliminary LOD of 50 copies per reaction. However, real-world clinical sample testing involves complex nucleic acid extraction procedures. To streamline the detection workflow, we evaluated an extraction-free sample preparation method using a commercially available lysis buffer compatible with the ERA reaction, obtained from GenDx Biotech (Suzhou, China). We optimized the lysis buffer volume by testing 1, 2, 5, and 10 mL on eight HPV-positive clinical samples. Detection rates improved with increasing lysis buffer volume: 3, 3, 6, and 6 positive samples were detected at 1, 2, 5, and 10 mL, respectively (Fig. 6A). Analysis of fluorescence fold change showed that samples 1, 6, and 8 remained positive with minimal variation across volumes, while samples 4 and 7 stayed negative (Fig. 6C). In contrast, increasing the lysis buffer volume converted samples 2, 3, and 5 to positive detection, with fold increases of 4.99, 9.95, and 10.14 for sample 2; 2.20, 5.04, and 7.39 for sample 3; and 5.48, 6.54, and 6.62 for sample 5 with 2, 5, and 10 mL, respectively (Fig. 6C). Notably, volumes above 5 mL did not further enhance detection, indicating 5 mL as the optimal lysis buffer volume.

Despite this optimization, two positive samples yielded false-negative results (Fig. 6A), indicating a need for further refinement of the lysis conditions. The lysis buffer containing Chelex-100 resin can reduce the inhibition caused by nasopharyngeal swabs in SARS-CoV-2 detection (30, 31). We, therefore, added Chelex-100 resin to the 5 mL lysis buffer to evaluate its effect on HPV detection in vaginal swabs. For the two previously undetected positive samples, the addition of Chelex-100 resin at gradient concentrations (0%, 10%, 20%, and 40%) enhanced detection signals (Fig. 6B). Analysis of fluorescence fold change showed that increasing the concentrations of Chelex-100 resin converted samples 4 and 7 to positive detection, with fold increases of 2.78, 6.11, and 6.14 for sample 4, and 2.65, 5.57, and 5.22 for sample 7, with 10%, 20%, and 40% Chelex-100, respectively (Fig. 6D). For samples that were already positive in the direct lysis buffer, increasing the Chelex-100 concentration did not alter the detection outcome, and the fluorescence fold change remained largely unchanged (Fig. 6D). These results indicate that the addition of Chelex-100 resin to the direct lysis buffer improves the detection of HPV-positive cervical swab samples, with 20% Chelex-100 resin meeting preliminary requirements.

### Clinical sample consistency validation using a low-cost lateral flow assay

**Fig 7:**
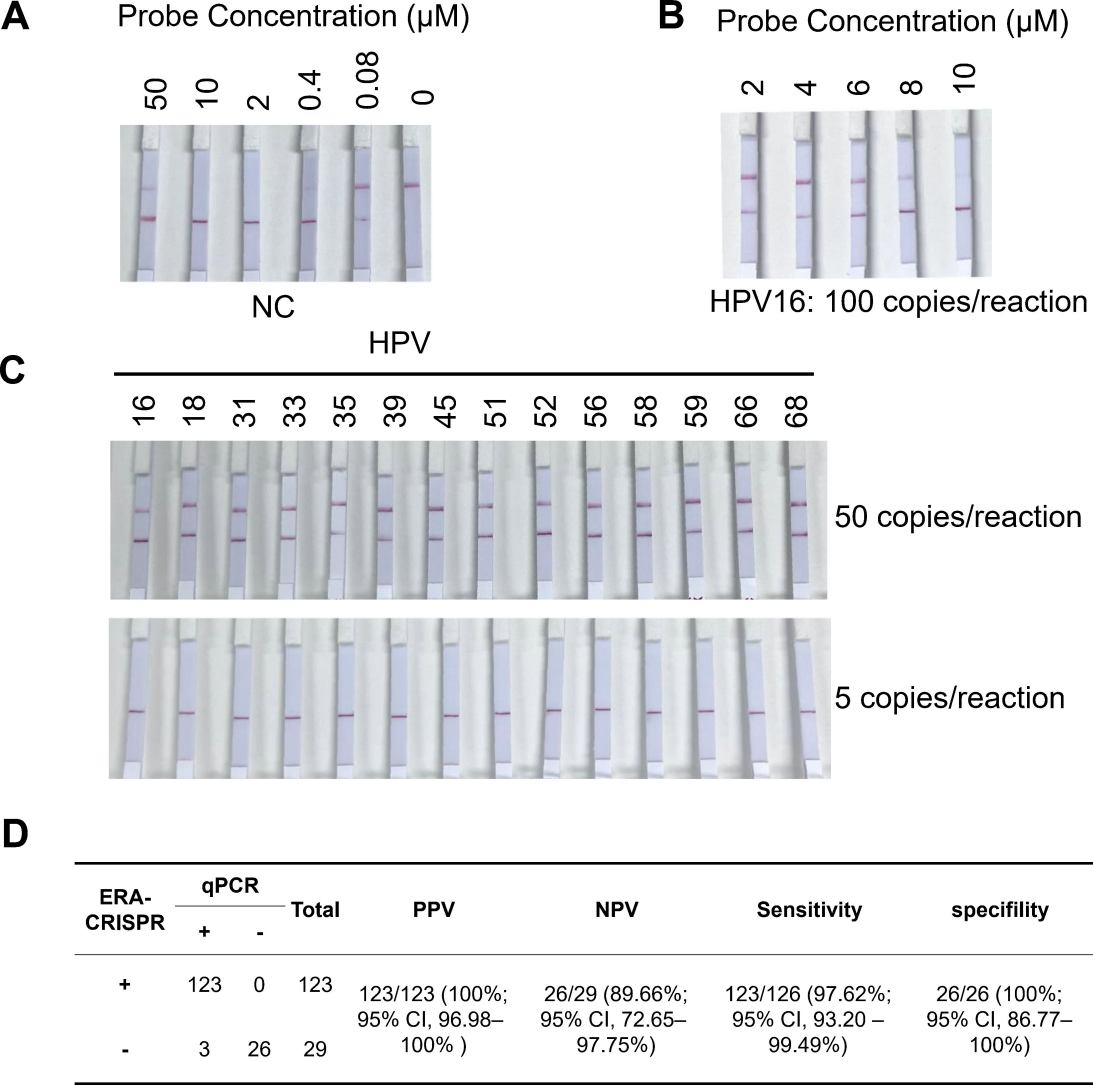
Testing results of the 14 HR-HPV in clinical samples. (**A**) Optimization of single-stranded probe concentrations (0, 0.08, 0.4, 2, 10, and 50 μM) in the CRISPR lateral flow detection system using negative control (NC) samples. (**B**) Sensitivity testing of different probe concentrations (2, 4, 6, 8, and 10 μM) using HPV16 reference standards. (**C**) LOD assessment for each HR-HPV type using a multiplex CRISPR assay with 5 and 50 copies per reaction. (**D**) Comparative analysis of RT-qPCR and the CRISPR-based lateral flow assay. Summary of diagnostic performance metrics, including positive predictive agreement (PPA), negative predictive agreement (NPA), sensitivity, and specificity, for the CRISPR-based detection system across clinical samples. The 95% confidence intervals were calculated using the Clopper-Pearson exact method.

The optimization process described above was based on fluorescence detection, which requires expensive equipment and limits the scalability of HPV testing. To improve cost-efficiency and expand testing coverage, we validated the performance of the 14 HR-HPV detection system using a low-cost lateral flow assay. Since the probe concentration in the CRISPR system affects the sensitivity of the lateral flow assay, we first optimized the probe concentration. We initially tested a range of probe concentrations to identify the threshold at which false-positive signals occur. False positives were observed when the probe concentration was below 0.4 μM or above 50 μM, whereas no false-positive signals were detected within the 2–10 μM range (Fig. 7A). We then assessed how different probe concentrations within this range affect the sensitivity of the detection system using the HPV16 plasmid. The results showed that concentrations of 2 or 4 μM yielded positive results. However, as the concentration increased to 6 or 8 μM, the signal intensity of the test bands gradually weakened. At 10 μM, the test result turned negative. Taken together, we found that a probe concentration of 2 or 4 μM provided the highest sensitivity for the lateral flow assay (Fig. 7A and B). To further evaluate the detection limit of the lateral flow assay, we compared it with the fluorescence-based method using corresponding reference standards. The results demonstrated that for all 14 HR-HPV types, a viral load of as low as 50 copies per reaction could still be reliably detected using the lateral flow assay (Fig. 7C).

For clinical validation, the probe concentration in the CRISPR lateral flow assay was standardized at 2 μM. Among 152 clinical samples, 126 were HR-HPV positive, including 90 single-genotype and 36 multiple-genotype infections, as confirmed by qPCR (Table S3). Our ERA-CRISPR system showed only three discrepancies were observed, both false negatives. Statistical analysis demonstrated robust performance of the assay, with a positive predictive value (PPV) of 100.0% (123/123; 95% CI, 96.98%–100%), negative predictive value (NPV) of 89.66% (26/29; 95% CI, 72.65%–97.75%), sensitivity of 97.62% (123/126; 95% CI, 93.20%–99.49%), and specificity of 100.0% (26/26; 95% CI, 86.77%–100%) (Fig. 7D; Fig. S2). In addition, ROC analysis based on grayscale intensity extracted from the lateral flow strips further confirmed the excellent diagnostic performance of the assay, yielding an area under the curve (AUC) of 0.996 (95% CI, 0.991%–1.00%) (Fig. S4). Collectively, these results confirm that the established CRISPR-based detection system enables reliable and accurate screening of 14 HR-HPV types in clinical samples.

## DISCUSSION

Persistent infection with HR-HPV is the primary cause of cervical cancer. Given the progressive nature of cervical cancer development, broad-coverage HR-HPV detection is critical for the early identification and management of high-risk HPV infections, ultimately reducing the incidence and mortality of cervical cancer. In this study, we developed and optimized a convenient, cost-effective, sensitive, and reliable CRISPR-based lateral flow assay (LFA) for the highly sensitive detection of HR-HPV in clinical samples.

In this study, we established a non-genotyping detection system targeting the 14 WHO-recommended HR-HPV types using mixed primer pools and mixed crRNA pools. While single crRNA assays achieved a detection limit of 10 copies per reaction (Fig. 2), the mixed crRNA pool failed to reach the same sensitivity (Fig. 5), suggesting that multiplexing of crRNAs compromises assay performance. Several mechanisms may account for this observation. First, when multiple crRNAs with diverse sequences are present, they compete for a limited number of Cas12a binding sites. This competition reduces the proportion of active Cas12a–crRNA complexes, thereby diminishing overall cleavage activity. Second, intermolecular interactions among crRNAs, such as secondary structure formation (e.g., hairpins or crRNA–crRNA pairing), may hinder their efficient association with Cas12a. Additionally, non-specific weak binding between some crRNAs and non-target DNA could occupy Cas12a-binding sites, further diluting target-specific signal output. It is noteworthy that, in this study, we selected only the most effective crRNA for each individual genotype when constructing the crRNA pools. However, the so called second-best crRNAs may exhibit superior performance in multiplex pools due to reduced structural interference or more balanced competition for Cas12a binding. Future studies should, therefore, systematically evaluate alternative crRNA combinations and optimize pooling strategies, with the aim of improving multiplex HPV detection sensitivity and robustness.

In recent years, CRISPR-based technologies have been increasingly applied in molecular diagnostics. Several CRISPR-based HPV detection methods have been reported, targeting different sets of HPV types depending on the intended application. These include assays for two HR-HPV types (HPV16 and HPV18) (29, 32–41), nine HR-HPV types (HPV6, 11, 16, 18, 31, 33, 45, 52, 58), 13 HR-HPV types (42), and 14 HR-HPV types (43). Although both our study and Yin et al. (43) focus on the same 14 HR-HPV genotypes, the two approaches differ substantially in workflow design, signal acquisition, and testing strategy. Specifically, Yin et al. (43) require a standalone DNA extraction and purification step, whereas our assay integrates an optimized direct lysis–amplification workflow by incorporating Chelex-100 resin into the lysis buffer to improve cellular disruption and nucleic acid release, resulting in enhanced analytical sensitivity and significantly streamlined operation. In addition, while Yin et al. (43) rely on fluorescence signal detection using fluorescence readers, our system employs lateral flow strips (LFS) for visual readout, eliminating equipment dependency and offering advantages in cost, portability, ease of interpretation, and point-of-care adaptability. Moreover, Yin et al. (43) rely on multi-well multiplex reactions to achieve HPV genotyping, whereas our system adopts a single-well, non-genotyping screening strategy, reporting positivity upon the detection of any of the 14 HR-HPV types, thereby facilitating rapid risk stratification for large-scale screening. This non-genotyping design is primarily based on two considerations. First, large-scale epidemiological studies have shown that among 2,728,321 women, 13.12% were infected with HR-HPV (44). From a cost-effectiveness perspective, performing an initial total HR-HPV screening followed by genotyping only for HR-HPV-positive cases could effectively reduce unnecessary testing expenses in low-income countries or regions while ensuring targeted screening for high-risk populations and optimizing resource allocation. Second, this approach reflects an inherent limitation of CRISPR-based assays: reporter probe cleavage is non-specific, making multiplexed genotyping within a single reaction technically challenging. Previous studies have demonstrated multi-target pathogen detection; for example, Yu et al. achieved HPV16/18 genotyping by exploiting the distinct cleavage activities of Cas12a (targeting single-stranded DNA) and Cas13 (targeting single-stranded RNA) with differentially labeled reporters (40), and Xu et al. applied microfluidic chips for genotyping multiple HPV types (45). However, extending such strategies to all 14 HR-HPV types would require separate reaction wells for each type, substantially increasing both costs and operational complexity compared with RT-PCR assays. Nonetheless, developing cost-effective and streamlined approaches for comprehensive HR-HPV genotyping without significantly increasing assay complexity remains an important direction for future research.

In nucleic acid detection, sample extraction and preparation represent critical yet often labor-intensive steps. The adoption of extraction-free strategies offers a promising means to streamline workflows and improve testing efficiency. However, in the context of genital tract microbiota detection, vaginal swab samples pose particular challenges, as their complex composition can inhibit amplification and cleavage reactions, thereby limiting the feasibility of extraction-free approaches. Although extensive studies and investments in SARS-CoV-2 testing have demonstrated the practicality of extraction-free workflows for rapid, large-scale screening (46), these strategies are not universally transferable to all pathogen targets. In our study, the limit of detection (LOD) for plasmid reference samples reached as low as 50 copies per reaction (Fig. 5). However, when applied to cervical/vaginal swab specimens, detection sensitivity decreased substantially (Fig. 6). This discrepancy is likely attributable to the higher complexity of vaginal samples compared with nasopharyngeal swabs used in SARS-CoV-2 testing, where inhibitory components are less prevalent. These findings highlight that the applicability of extraction-free approaches is highly sample type–dependent, and optimizing sample preparation methods tailored to specific pathogens is essential for ensuring both sensitivity and reliability. Furthermore, previous studies have shown that the incorporation of Chelex-100 into rapid lysis buffers can mitigate inhibitory effects and significantly improve detection sensitivity in nasopharyngeal swab-based SARS-CoV-2 assays (30, 31). Consistent with this, our results demonstrated that supplementing the direct amplification lysis buffer with Chelex-100 substantially enhanced HPV detection performance in vaginal swab samples (Fig. 6). Taken together, our findings and previous studies indicate that the Chelex-100-based extraction method offers a rapid and simple alternative to conventional nucleic acid purification techniques. Beyond its simplicity and time efficiency, this approach also provides a significant cost advantage. Previous reports have shown that the per-sample extraction cost can be reduced from approximately €3.46 when using the QIAamp kit to as low as €0.02 with the Chelex-100 method (47). While Chelex-100 resin improves HPV detection in our direct lysis buffer system, several practical limitations remain for large-scale clinical implementation. Manual aliquoting of Chelex-100 into individual samples is labor-intensive, and batch-to-batch variation may affect extraction consistency. In terms of stability, although the lysis buffer itself can be stored at room temperature for extended periods, Chelex-100 requires long-term storage under dry conditions. The stability of the complete lysis buffer containing 20% Chelex-100 is currently unknown, as the resin’s stability may be compromised once incorporated into the aqueous buffer. Therefore, future studies are needed to systematically evaluate the stability of Chelex-100 in the lysis buffer. Such studies would clarify whether the resin should be pre-mixed with the lysis buffer for convenience or added immediately before detection, and if pre-mixed, how storage conditions should be optimized to ensure consistent performance. Addressing these aspects will be essential for translating this extraction-free approach into routine, large-scale clinical workflows.

Here, we adopted a sequential strategy for the detection of 14 HR-HPV types, in which nucleic acid amplification and CRISPR-based endpoint detection were performed as two separate steps. This design required transferring amplification products into a distinct system for CRISPR detection. However, such an approach inevitably increases workflow complexity and introduces the risk of aerosol contamination during sample transfer, potentially leading to false-positive outcomes, which represents a limitation of our method. To reduce contamination risk, amplification and high-amplicon workflows should be spatially isolated from reagent preparation, with minimal tube opening and strict surface decontamination using hypochlorite, DNA-degrading agents, and UV irradiation. Each batch should include no-template controls, supported by periodic environmental monitoring. Incorporating a dUTP/UNG system can additionally prevent carryover contamination, provided that polymerase tolerance to dUTP is validated to avoid reduced amplification efficiency. Despite these measures reducing aerosol contamination to some extent, further mitigation requires source control through a fully closed, single-reaction system. Integrating nucleic acid amplification with CRISPR detection into a single “one-pot” reaction offers the most effective solution, as it streamlines the workflow and minimizes contamination risks associated with tube opening and sample transfer. This design is particularly advantageous for low-resource settings and point-of-care testing. However, most current one-pot formats still exhibit reduced analytical sensitivity compared with the conventional two-step workflow (48). Several factors may account for this reduction. First, during target recognition, the cis-cleavage activity of CRISPR/Cas12a can degrade amplified DNA templates, thereby interfering with amplification efficiency (49). Second, its trans-cleavage activity may indiscriminately degrade single-stranded primers in the recombinase polymerase amplification (RPA) system, further compromising amplification performance (22, 49). Despite these challenges, overcoming the limitations of one-pot CRISPR detection is critical for expanding its utility in molecular diagnostics. In recent years, substantial progress has been made in optimizing one-pot detection strategies (50–57). For physical compartmentalization, studies have immobilized Cas12a components on the tube wall or cap (51–54, 58) or employed microfluidic chips to spatially separate amplification and CRISPR reactions (55, 56), thereby minimizing interference with amplification. In terms of reagent optimization, reducing Cas12a concentration has been shown to alleviate inhibition of RPA, but often at the cost of reduced sensitivity and prolonged reaction time (59). Additionally, Lin et al. (57) improved detection sensitivity by two orders of magnitude by adding glycerol to the one-pot system. Lu et al. (60) optimized crRNA and found that a suboptimal PAM sequence performed better than the standard PAM sequence in the one-pot assay although it still exhibited reduced sensitivity compared to the conventional two-step method. Despite these advances, current optimization strategies continue to face limitations, including the need for additional centrifugation steps, insufficient sensitivity, and increased costs. To overcome these challenges, future studies should integrate current optimization strategies with advanced approaches to further refine the detection system for 14 HR-HPV types, thereby enhancing sensitivity, reliability, and applicability in molecular diagnostics. In particular, the development of high-activity Cas12a variants, universal and compatible buffer formulations, and incorporation of signal amplification strategies such as cascade reactions could provide further improvements.

Beyond its technical advantages, the ERA-CRISPR assay we developed for HR-HPV detection offers notable cost savings. By detecting 14 HR-HPV types in a single reaction using a direct amplification–lysis system, the assay minimizes both reagent and operational costs. The main expenses—ERA reagents, Cas12a cleavage reagents, and lateral flow strips—amount to approximately $2.5 per test, comparable to previously reported CRISPR-based assays ($2–5 per test) (61–63). The required equipment is highly portable, needing only a constant-temperature heating device, which substantially reduces infrastructure investment. In contrast, commercial HPV qPCR kits, while relatively inexpensive in reagents, rely on costly real-time PCR instruments, resulting in per-test costs of $10–20, largely due to thermal cycler maintenance ($5,000–10,000/year) and fluorescence detection modules that account for over 75% of hardware costs (61, 64). Moreover, CRISPR-based assays have been reported to reduce total testing costs by up to 80% in resource-limited settings (65), suggesting that the ERA-CRISPR assay may hold even greater application potential in these regions.

Although the assay demonstrated robust analytical and clinical performance, several limitations should be acknowledged. First, we did not systematically assess the long-term stability of the Cas12a enzyme, a key reagent in the ERA-CRISPR workflow. Although the manufacturer indicates that Cas12a is stable for up to 12 months at −20°C, such cold-chain requirements may increase logistical burdens, especially in resource-limited settings. Lyophilization may help overcome this limitation, as previous studies have shown that Cas12 enzymes retain comparable activity before and after lyophilization (21), highlighting its potential for improving reagent stability; future work will focus on optimizing a lyophilized Cas12a system to enhance field applicability. In addition, although the samples were collected consecutively, the study population primarily comprised individuals undergoing gynecological evaluation rather than routine screening, which resulted in a higher HR-HPV prevalence compared with that reported in general screening populations (44). Among 152 samples, 126 were HR-HPV positive, including 90 single-genotype and 36 multiple-genotype infections (Table S3). The high proportion of multiple infections (28.6%) may be easier to detect, potentially overestimating assay performance for single-genotype infections. Notably, HPV31, HPV45, and HPV66 were only observed in mixed infections, limiting representation of certain types. Future studies with expanded sample size and targeted collection of single-genotype infections for these underrepresented types will allow a more comprehensive evaluation of assay performance across all 14 HR-HPV types.

In summary, this study optimized a detection strategy for 14 HR-HPV types by integrating extraction-free technology, enzyme-mediated isothermal rapid amplification, and CRISPR/Cas12a-based detection. The proposed method exhibits simplicity, rapidity, affordability, and high accuracy, making it a promising approach for HPV screening. Although there are still limitations in operational simplification, further refinements will enhance its accessibility, particularly in low-resource settings, thereby facilitating early detection and contributing to cervical cancer prevention.

## SUPPLEMENTAL MATERIAL

Supplemental material spectrum.03036-25-s0001.docx Fig. S1 to S4; Tables S1 to S3.

ASM does not own the copyrights to Supplemental Material that may be linked to, or accessed through, an article. The authors have granted ASM a non-exclusive, world-wide license to publish the Supplemental Material files. Please contact the corresponding author directly for reuse.
