# Supplementary material for: Development and clinical validation of an ERA-CRISPR/Cas12a assay for the rapid detection of 14 high-risk HPV types
Source: Microbiol Spectr. 2025 Dec 26;14(2):e03036-25. doi: 10.1128/spectrum.03036-25 (PMC12889081; doi:10.1128/spectrum.03036-25)
Supplement: Supplemental material — Fig. S1 to S4; Tables S1 to S3. [file spectrum.03036-25-s0001.docx]

**Development and Clinical Validation of an ERA-CRISPR/Cas12a Assay for the Rapid Detection of 14 High-Risk HPV Types**

**Running title:** Rapid Detection of 14 HR-HPV Types by ERA-CRISPR

Zhijie Wang^1,2, #^, Ting Hu^1,2, #^, Wanxin Liu^1,2^, Hu Zhou^1,2^, Xinyi Lv ^1,2^, Hui Li^3^, Xuemeng Li^3^, Xiaoyuan Huang^1,2, *^, Liang He^1,2, *^

^1^Department of Obstetrics and Gynecology, Tongji Hospital, Tongji Medical College, Huazhong University of Science and Technology, 430030, Wuhan, Hubei, China.

^2^National Clinical Research Center for Obstetrics and Gynecology, Cancer Biology Research Center (Key Laboratory of the Ministry of Education), Tongji Hospital, Tongji Medical College, Huazhong University of Science and Technology, Wuhan 430030, Wuhan, Hubei, China.

^3^Wuhan Kandwise Biotechnology, Inc. Wuhan, Hubei, China.

# These authors contributed equally to this work.­

*To whom correspondence should be addressed: Xiaoyuan Huang, Email: huangxy@tjh.tjmu.edu.cn; Liang He, Email: whuheliang@outlook.com.

**Supporting Information**

**
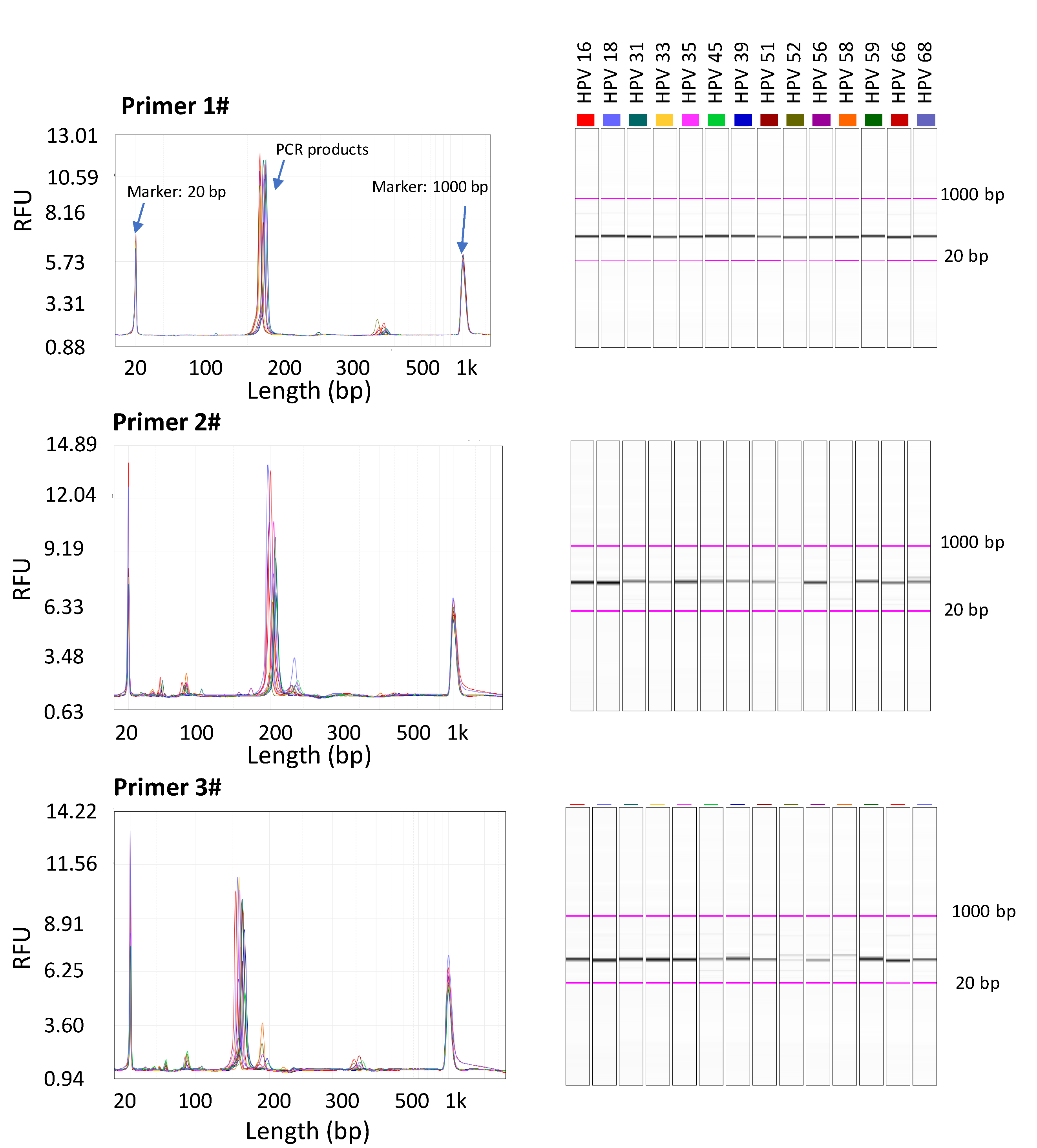
**

**Fig. S1. Capillary electrophoresis validation of candidate primers for 14 high-risk HPV types.** The top, middle, and bottom panels show the amplification performance of the first, second, and third primer sets, respectively. The amplification efficiency of these primers was evaluated using the Qsep100 capillary electrophoresis system. Different colors represent different HPV genotypes.

**
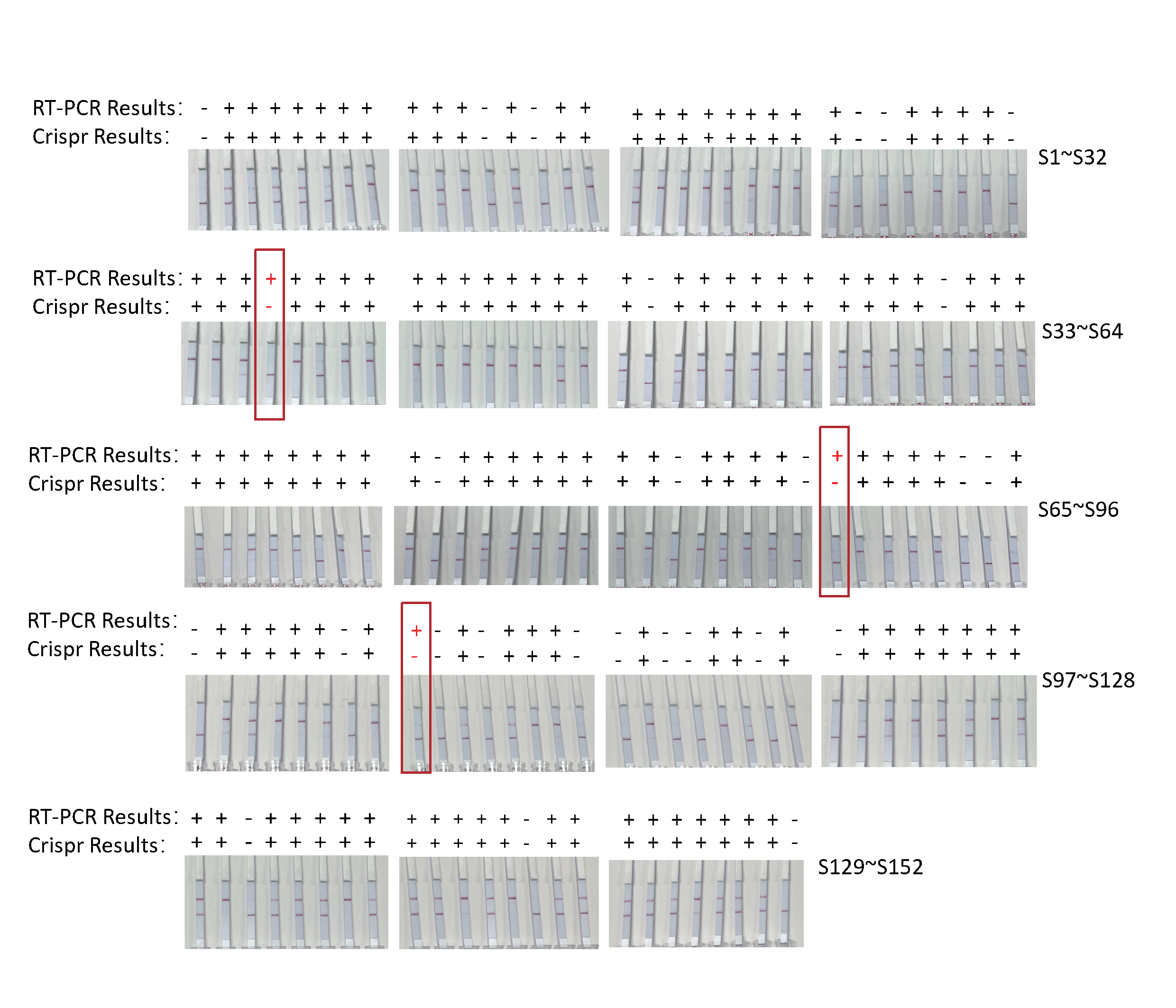
**

**Fig. S2. Clinical validation of the ERA-CRISPR lateral flow assay system.** A total of 152 clinical samples were analyzed using the ERA-CRISPR lateral flow assay system, and the results were compared with RT-PCR results obtained from the HPV genotyping kit (Bioperfectus Technologies, Jiangsu, China). Samples with discrepant results between the two methods are highlighted with red boxes.


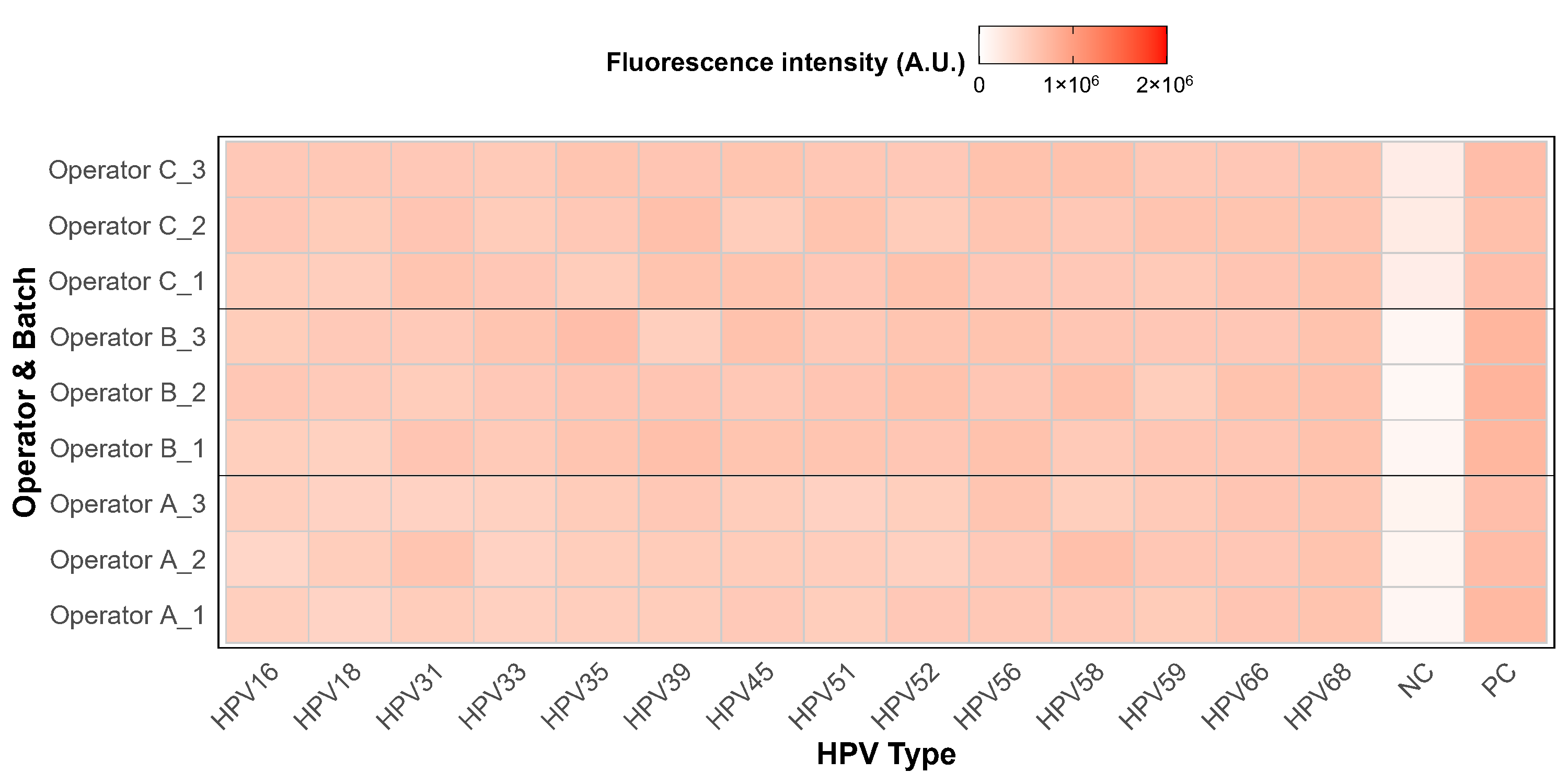


**Fig. S3. Reproducibility assessment across operators and runs.**Heatmap showing the reproducibility of the ERA-CRISPR assay using limit-of-detection (LOD) samples (50 copies per reaction) for all 14 HR-HPV types. Three operators (Operator A, B, and C) each performed three batches. For every HPV type, positive samples yielded consistent fluorescence signals across operators and replicates, while the negative control (NC) remained negative. The uniform detection pattern across all conditions demonstrates high inter-operator and inter-run reproducibility of the assay.


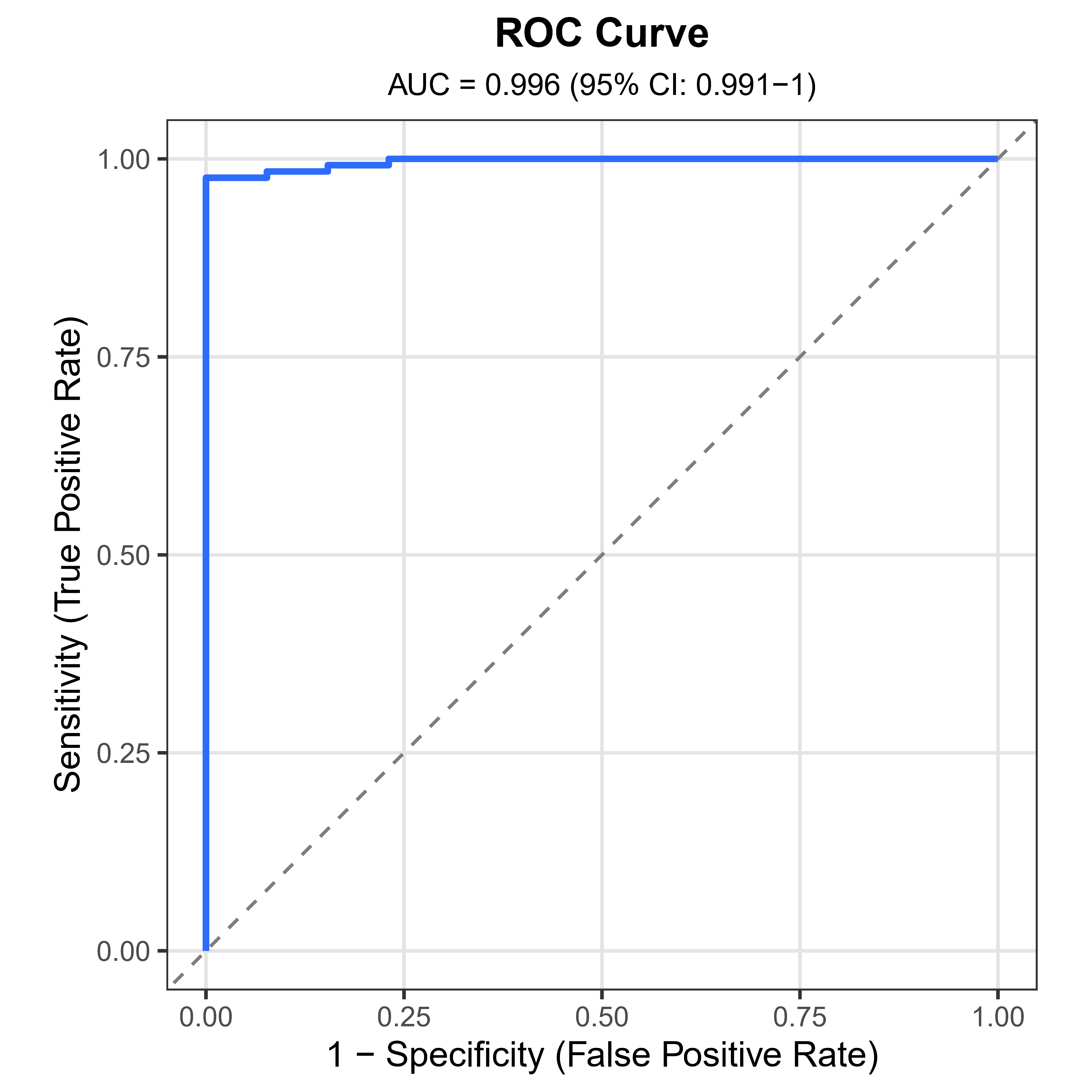


**Fig. S4.** Receiver operating characteristic (ROC) curve evaluating the diagnostic performance of the ERA-CRISPR assay for detecting 14 HR-HPV types in 152 clinical samples, using qPCR as the reference standard. ROC analysis was based on continuous grayscale intensity values quantified from lateral flow strips using ImageJ software. The assay showed an AUC of *0.996* (95% CI: *0.991–1*).

**Table S1.** Final sequences of selected primers targeting 14 HR HPV types.

| Primer | Sequence (5′→3′) |
| --- | --- |
| 16-F | ACAGGGCCACAATAATGGCATTTGTTGG |
| 18-F | ACAGGGTCATAACAATGGTGTTTGCTGG |
| 31-F | TCAGGGACACAATAATGGTATTTGTTGG |
| 33-F | ACAAGGTCATAATAATGGTATTTGTTGG |
| 35-F | ACAAGGCCATAATAATGGTATTTGTTGG |
| 39-F | CCAGGGCCACAACAATGGTATATGTTGG |
| 45-F | CCAGGGCCATAACAATGGTATTTGTTGG |
| 51-F | GCAGGGTCACAATAATGGCATTTGCTGG |
| 52-F | GCAGGGCCACAATAATGGCATATGTTGG |
| 56-F | CCAAGGCCATAATAATGGCATTTGCTGG |
| 58-F | ACAAGGTCATAACAATGGCATTTGCTGG |
| 59-F | TCAGGGTTTAAACAATGGTATATGTTGG |
| 66-F | ACAGGGCCATAATAATGGCATATGCTGG |
| 68-F | ACAGGGACACAACAATGGTATTTGTTGG |
| 16-R | GAAAAATAAACTGTAAATCATATTCCTC |
| 18-R | GAAAAATAAACTGCAAATCATATTCCTC |
| 31-R | GAAATATAAATTGTAAATCAAATTCCTC |
| 33-R | GAAAAACAAACTGTAGATCATATTCTTC |
| 35-R | GAAAAATAAACTGTAAATCATATTCTTC |
| 39-R | GAAATATAAATTGTAAATCATACTCCTC |
| 45-R | GAAAAATAAACTGTAAATCATATTCCTC |
| 51-R | GAAAAATAAATTGCAATTCATACTCTTC |
| 52-R | GAAAAATAAATTGTAAATCGAATTCCTC |
| 56-R | GAAAAACAAATTGTAATTCATATTCCTC |
| 58-R | GAAAAACAAACTGTAAGTCATATTCTTC |
| 59-R | GAAATATAAACTGCAAATCAAATTCCTC |
| 66-R | GAAACACAAACTGTAGTTCATATTCCTC |
| 68-R | GAAATATAAATTGCAAATCATATTCCTC |

**Table S2.** Final sequences of selected crRNAs targeting 14 HR HPV types.

| Primer | Sequence (5′→3′) |
| --- | --- |
| 16crRNA-3 | uaauuucuacuaaguguagauAGGAGUACCUACGACAUGGGGAG |
| 18crRNA-1 | uaauuucuacuaaguguagauGUAGCAUCAUAUUGCCCAGGUAC |
| 31crRNA-2 | uaauuucuacuaaguguagauAGACAUGGUGAGGAAUUUGAUUU |
| 33crRNA-2 | uaauuucuacuaaguguagauUGCACACAAGUAACUAGUGACAG |
| 35crRNA-1 | uaauuucuacuaaguguagauUAUGUACUGUCACUAGAAGACAC |
| 39crRNA-2 | uaauuucuacuaaguguagauAGGAAUAUACCAGGCACGUGGAG |
| 45crRNA-2 | uaauuucuacuaaguguagauAGCAGUAUAGUAGACAUGUGGAG |
| 51crRNA-1 | uaauuucuacuaaguguagauACUAUUAGCACUGCCACUGCUGC |
| 52crRNA-3 | uaauuucuacuaaguguagauUAUGUGCUUUCCUUUUUCACCUC |
| 56crRNA-1 | uaauuucuacuaaguguagauGUGCAUCAUAUUUACUUAACUGU |
| 58crRNA-2 | uaauuucuacuaaguguagauAGGAAUAUGUACGUCAUGUUGAA |
| 59crRNA-2 | uaauuucuacuaaguguagauAAGAAUAUGCCAGACAUGUGGAG |
| 66crRNA-1 | uaauuucuacuaaguguagauGCUGCAUUAAUAGUCAUGUUGGU |
| 68crRNA-2 | uaauuucuacuaaguguagauCUUUGUCUACUACUACUGAAUCA |

**Table S3.** Distribution of HPV genotypes in 152 clinical samples

| **HPV Genotype(s)** | **Sample ID** | **Total** |
| --- | --- | --- |
| (-) | S1, S12, S14, S26, S27, S32, S50, S61, S74, S83, S88, S94, S96, S97, S103, S106, S108, S112, S113, S115, S116, S119, S121, S131, S142, S152 | 26 |
| HPV16 | S3, S9, S16, S19, S43, S53, S55, S57, S65, S66, S69, S77, S87, S89, S91, S100, S101, S128, S149 | 19 |
| HPV18 | S15, S28, S29, S39, S67, S68, S120, S133 | 8 |
| HPV33 | S47, S71, S76, S104, S123, S125, S145 | 7 |
| HPV35 | S44 | 1 |
| HPV39 | S5, S18, S23, S111, S141 | 5 |
| HPV51 | S37, S70, S102, S109, S140 | 5 |
| HPV52 | S6, S10, S13, S20, S22, S24, S33, S36, S54, S72, S79, S85, S124, S132, S138, S144 | 16 |
| HPV56 | S4, S8, S46, S62, S105 | 5 |
| HPV58 | S2, S31, S34, S35, S49, S56, S73, S78, S81, S86, S93, S95, S114, S117, S118, S127, S137 | 17 |
| HPV59 | S130 | 1 |
| HPV68 | S60, S63, S129, S135, S136, S143 | 6 |
| HPV16, 18 | S52 | 1 |
| HPV16, 18, 52, 59, 68 | S82 | 1 |
| HPV16, 31, 56 | S42 | 1 |
| HPV16, 33, 68 | S151 | 1 |
| HPV16, 39 | S64 | 1 |
| HPV16, 45 | S7 | 1 |
| HPV16, 52 | S25, S30, S59 | 3 |
| HPV16, 52, 59 | S51 | 1 |
| HPV16, 56, 68 | S40 | 1 |
| HPV16, 58 | S58, S98 | 2 |
| HPV18, 31, 52, 58, 68 | S45 | 1 |
| HPV18, 52 | S110, S126 | 2 |
| HPV18, 68 | S92 | 1 |
| HPV33, 58 | S17 | 1 |
| HPV35, 52 | S48, S148 | 2 |
| HPV35, 58, 66 | S122 | 1 |
| HPV39, 52 | S107 | 1 |
| HPV39, 59 | S147 | 1 |
| HPV39, 68 | S99 | 1 |
| HPV51, 52 | S84 | 1 |
| HPV51, 56 | S146 | 1 |
| HPV51, 59 | S90, S134 | 2 |
| HPV52, 58 | S41 | 1 |
| HPV52, 59 | S21, S75 | 2 |
| HPV56, 58 | S139, S150 | 2 |
| HPV56, 59 | S11, S38 | 2 |
| HPV56, 66 | S80 | 1 |
